# Applications of the Microalgae *Chlamydomonas* and Its Bacterial Consortia in Detoxification and Bioproduction

**DOI:** 10.3390/life14080940

**Published:** 2024-07-27

**Authors:** María J. Torres, Carmen M. Bellido-Pedraza, Angel Llamas

**Affiliations:** Department of Biochemistry and Molecular Biology, Campus de Rabanales and Campus Internacional de Excelencia Agroalimentario (CeiA3), Edif. Severo Ochoa, University of Córdoba, 14071 Córdoba, Spain; b22bepec@uco.es

**Keywords:** algal–microbial consortia, microalga, *Chlamydomonas*, bioremediation, biofertilization, high-value added products

## Abstract

The wide metabolic diversity of microalgae, their fast growth rates, and low-cost production make these organisms highly promising resources for a variety of biotechnological applications, addressing critical needs in industry, agriculture, and medicine. The use of microalgae in consortia with bacteria is proving valuable in several areas of biotechnology, including the treatment of various types of wastewater, the production of biofertilizers, and the extraction of various products from their biomass. The monoculture of the microalga *Chlamydomonas* has been a prominent research model for many years and has been extensively used in the study of photosynthesis, sulphur and phosphorus metabolism, nitrogen metabolism, respiration, and flagellar synthesis, among others. Recent research has increasingly recognised the potential of *Chlamydomonas*–bacteria consortia as a biotechnological tool for various applications. The detoxification of wastewater using *Chlamydomonas* and its bacterial consortia offers significant potential for sustainable reduction of contaminants, while facilitating resource recovery and the valorisation of microalgal biomass. The use of *Chlamydomonas* and its bacterial consortia as biofertilizers can offer several benefits, such as increasing crop yields, protecting crops, maintaining soil fertility and stability, contributing to CO_2_ mitigation, and contributing to sustainable agricultural practises. *Chlamydomonas*–bacterial consortia play an important role in the production of high-value products, particularly in the production of biofuels and the enhancement of H_2_ production. This review aims to provide a comprehensive understanding of the potential of *Chlamydomonas* monoculture and its bacterial consortia to identify current applications and to propose new research and development directions to maximise their potential.

## 1. Introduction

Microalgal cells are photosynthetic unicellular organisms that play a crucial role in global ecosystems, contributing to over half of the Earth’s total photosynthetic activity and forming the backbone of the food chain [1]. Despite their prevalence in aquatic environments, microalgae thrive in diverse habitats, including the cold and irradiated poles, the subterranean environment of densely packed rhizospheres, and even within animal tissues in coral reefs [2]. Microalgae share a common evolutionary origin that can be traced back to a primary endosymbiotic event involving a cyanobacterium, which eventually evolved into the plastid. Subsequently, this plastid lineage expanded through secondary and tertiary endosymbiosis [3]. Microalgae play a crucial role in supporting ecosystems; however, they can also disrupt them through algal blooms, posing significant ecological and health risks [4]. Microalgae have demonstrated a wide range of metabolic capabilities and possess several unique properties that make them highly valuable for scientific studies [5]. Furthermore, the use of microalgae in diverse biotechnological applications has significantly increased in the last century. Emerging applications of microalgae include the production of biomaterials as alternatives to fossil-based materials, including biofertilizers, biostimulants, biopesticides, and energy sources such as biodiesel, bioethanol, and biogas [6]. Microalgae are a source of bioactive compounds, including essential amino acids, polyunsaturated fatty acids, and antioxidants, which have been shown to positively impact nutrition and health [7]. Microalgae are considered a sustainable resource due to their ability to be used in detoxification processes, converting waste into valuable products within a circular economy model [8]. Consequently, microalgae are gaining global attention for their significant ecological importance.

*Chlamydomonas* is a genus of single-celled biflagellate green microalgae that are mostly found in freshwater, but some species can also be found in habitats as diverse as salt water, soil, and snow. Taxonomically, the genus *Chlamydomonas* comprises more than 500 species [9]. *Chlamydomonas reinhardtii* is undoubtedly the most studied species of the *Chlamydomonas* genus. *C. reinhardtii* was isolated in 1945 from the soil of a potato field in Massachusetts, USA [10]. Since then, it has been used to investigate a range of research topics, including photosynthesis, respiration, sulphur and phosphorus metabolism, nitrogen metabolism, amino acid and metal metabolism, biosynthetic pathways of starch, carotenoids, lipids, glycerolipids, heme groups, and chlorophyll. Additionally, it has been used to study other fundamental aspects such as the function of chaperones, proteases, flagella biogenesis, thioredoxins, and responses to various stress conditions [9]. Advances in various gene-editing techniques, such as CRISPR-Cas9 in *Chlamydomonas,* represent significant progress in addressing basic research inquiries and biotechnological applications [11]. All this demonstrates that *Chlamydomonas* has been intensively used in basic research; however, the practical applications of *Chlamydomonas* for certain biotechnological purposes are not as numerous. Nevertheless, there has been a noticeable increase in interest in its potential biotechnological use in recent years [12].

Microalgae have been isolated from their natural habitats, with many proving challenging to cultivate axenically. This reliance on other microorganisms likely stems from the long-term co-evolution between microalgae and their beneficial microbes [13]. In this sense, microalgae have the capability to establish symbiotic associations with a diverse array of organisms, including bacteria, fungi, plants, and animals [14]. The term “phycosphere” refers to the area where algal exudates affect neighbouring microorganisms [15]. In mutualistic relationships, microalgae typically provide their partner(s) with fixed carbon and oxygen in exchange for essential nutrients and molecules such as carbon dioxide (CO_2_), various vitamins, and nitrogen [16]. In addition, a wide range of molecules can be secreted, detected, and used by the interacting partners to establish a complex molecular dialogue. Understanding of the complexity and dynamics of many of these symbiotic relationships is still in its infancy [17]. Several studies have focused on analysing the phycosphere microbiota to identify and understand the important interactions between microalgae and bacteria in their natural habitats [18,19]. Consequently, researchers have characterised the phycosphere of several microalgae with significant industrial potential, such as *Chlamydomonas* and other green algae such as *Chlorella vulgaris, Scenedesmus sp., and Botryococcus braunii*. In one study, the phycosphere of *Chlamydomonas* was found to be predominantly inhabited by *Hyphomicrobiaceae* sp. (44%), *Mesorhizobium* sp. (15%), and *Rhizobium* sp. (12%) [20]. The genomes of *Chlamydomonas*-associated bacteria are likely to encode a wide range of enzymes and metabolic pathways that could be exploited for bioremediation applications, such as the degradation of pollutants or the sequestration of heavy metals. Further investigation of the *Chlamydomonas* microbiome and its biotechnological potential represents an exciting avenue for future research. The plant-growth-promoting bacterium *Methylobacterium* species can support *Chlamydomonas* growth when L-proline is the sole nitrogen source, creating a mutualistic relationship. This species-specific interaction involves a carbon–nitrogen exchange: the bacterium metabolises L-proline to produce ammonium (NH_4_^+^), which feeds *Chlamydomonas*, while the alga releases glycerol, a photosynthate that feeds the bacterium [21]. The algal-produced indole-3-acetic acid (IAA) has recently been suggested to play a role in mutualistic interactions between *Chlamydomonas* and *Methylobacterium* spp. When nitrogen is limited, *Chlamydomonas* synthesises IAA from L-tryptophan through a process mediated by L-amino acid oxidase (LAO1). The accumulation of this auxin in the environment stops algal cell division and chlorophyll degradation. *Methylobacterium* spp. can alleviate this inhibitory effect by metabolising the auxin for growth. The presence of the algae enhances the bacterial degradation of auxin, leading to reduced levels and allowing *Chlamydomonas* to resume growth. Given their presence in the rhizosphere, this chemical communication mediated by auxin production and degradation could influence plant health and potentially increase crop yields in sustainable agriculture [22]. The highly reactive H_2_O_2_ produced by LAO1 may help *Chlamydomonas* to avoid competitors and promote the growth of beneficial bacteria that specifically contain H_2_O_2_-detoxifying enzymes such as catalase. In support of this idea, H_2_O_2_-consuming bacteria are present in the natural habitat of the diatom *Amphiprora kufferathii*, which can eliminate the H_2_O_2_ produced by the algal cells [23].

Since its initial discovery, there has been a significant increase in the number of studies exploring the symbiotic relationships between *Chlamydomonas* and bacteria, mainly focusing on fundamental research [24,25]. However, to the best of our knowledge, there has never been a single review that summarises the potential of *Chlamydomonas*–bacteria consortia for important biotechnological tasks such as wastewater treatment, detoxification, biofertilizer production, biomass valorisation, and bioproduct production. Therefore, here we summarise and categorise these reports with the aim of highlighting the potential of *Chlamydomonas*–bacteria consortia to fulfil these tasks.

## 2. Microalgae Cultivation Methods

*C. reinhardtii* has shown an exceptional ability to adapt and thrive under almost all experimental conditions tested in heterotrophic, phototrophic, and mixotrophic cultivation [26]. In addition, the *Chlamydomonas* Sourcebook [27] provides a thorough overview of the major areas of research, historical background, physiology, and methodology related to *Chlamydomonas*. For the correct and efficient use of *Chlamydomonas*–bacteria co-cultures for any biotechnological purpose, it is essential to optimise the cultivation conditions and choose the appropriate cultivation method. A perfect microalgal cultivation system should be easy to manage, cost effective to build, have sufficient light exposure, facilitate efficient gas–liquid transfer, and have a minimal risk of contamination. Photobioreactors have emerged as a potentially sustainable method for removing wastewater contaminants while producing microalgal biomass [28]. Typically, reactor configurations used in microalgal–bacteria systems include suspended systems (open reactors and closed reactors) and attached systems (such as biofilm reactors and encapsulated microalgae). Therefore, we will now present the main cultivation methods available for use with microalgae, highlighting the specific characteristics for microalgae–bacteria cocultivation and providing examples of their biotechnological potential in each case.

Open reactors include natural ponds and lakes, as well as specially designed high-rate algal ponds (HRAPs), which are tanks or lagoons with a paddle wheel that circulates wastewater. HRAPs can be a cost-effective and environmentally friendly approach to wastewater treatment, as microalgae are efficient at absorbing nutrients such as nitrogen and phosphorus, as well as helping to remove organic and inorganic contaminants [29]. Unlike indoor laboratory-scale cultivation, outdoor algae cultivation using HRAPs is heavily influenced by various uncontrollable environmental factors such as seasonal changes and weather conditions [30]. The following are the generally accepted design parameters for algae–bacteria HRAPs: depths typically ranging from 0.2 to 1 m, depending on wastewater clarity for light penetration; horizontal water velocities between 0.09 and 0.3 m/s to ensure good mixing; hydraulic residence times (HRTs) from 3 to 15 days; and HRAP areas from 1000 to 50,000 m^2^ [31,32]. Numerous studies have reported the use of HRAPs in wastewater treatment, mainly focusing on genera such as *Scenedesmus* and *Chlorella* [33]. It has been reported that NH_4_^+^ can be evaporated and removed primarily by nitrification, followed by assimilation and denitrification, when anaerobically digested pig manure is treated with algal–bacterial HRAPs and taken up by the microalgal cell [34]. However, there are very few reports on the use of HRAPs with *Chlamydomonas*. An experimental pilot-scale wastewater treatment using HRAPs with *Chlamydomonas* sp. reported reductions of approximately 90% in biochemical oxygen demand (BOD), 65% in chemical oxygen demand (COD), 20% in total phosphorus, and 46% in total nitrogen [35]. A study on the detoxification of piggery effluent using HRAPs with *Chlamydomonas* sp. showed an average COD and total nitrogen removal efficiencies of 76% and 88%, respectively [36]. In another study using HRAPs with *Chlamydomonas* sp. to treat municipal wastewater, the average reductions in total suspended solids, biochemical oxygen demand, and total nitrogen were 63%, 98%, and 76%, respectively [37]. Open systems other than HRAPs are also used for wastewater treatment with microalgae, such as thin-layer cascade systems. These systems show great promise for the applications of microalgae cultures in wastewater treatment, as they are easily scalable from small to full scale [38].

Photobioreactors (PBRs) are closed systems used for the cultivation of microalgae and other phototrophic microorganisms. PBRs can be operated in any open space and provide excellent control of culture conditions with minimal risk of contamination. Numerous studies have used PBRs with *Chlamydomonas*, mainly for basic research purposes. Maximum biomass productivities in *C. reinhardtii* were investigated using two different PBRs: a torus-plane reactor and a cylindrical reactor. The research highlighted that optimising the design of *Chlamydomonas* PBRs requires the management of three key parameters: the specific illuminated area, the illuminated working volume fraction, and the mean value of the incident hemispherical photon flux density [39]. In addition, the optimisation of a pilot-scale photobioreactor (120 L) for *Chlamydomonas* showed that the design of the gas diffuser had a significant effect on biomass production [40]. A specially designed 110 L PBR for *C. reinhardtii* was constructed from polypropylene and transparent Plexiglas. It has 64 tubes arranged in an 8 × 8 square pitch cell, connected by U-bends, with a total length of 133 m [41].

However, PBRs are more expensive to manufacture and require the use of transparent materials such as glass and acrylic in their construction. PBRs are characterised by a narrow light path and a high ratio of illuminated surface area to volume, which maximises the capture and conversion of light energy [42]. PBR modules can be organised in various configurations such as horizontal, inclined, vertical, or spiral arrangements. Flat-plate and tubular photobioreactors are common designs due to their large illuminated areas [43]. Next, we present some recent studies that have led to PBR applications with significant biotechnological potential. The use of the phototactic response of *C. reinhardtii* to induce biomixing in PBR has been investigated. By exploiting this phototactic mechanism, *C. reinhardtii* can be stimulated to swim in opposite directions, allowing mixing and ensuring access to nutrients without additional energy cost. This approach could potentially replace mechanical stirring in certain circumstances, thereby increasing energy efficiency [44]. However, so far it has only been tested with small volumes (40 mL); its industrial application would require subsequent scaling up. A multi-scale modular PBR, Antares I, has been tested with *C. reinhardtii* and has shown improved biomass production compared to traditional flask systems. With Antares I, the estimated doubling time for *Chlamydomonas* culture was almost half that reported for culture using similar media and light conditions [45]. In the Antares I system, several small devices are combined in a modular framework to feed and maintain the central growth vessels (1.7 and 7.5 L). The volumetric oxygen mass transfer coefficient was determined to be 21.89 h^−1^ at an air flow rate of 50 µL/s. The system described controls temperature, light intensity, CO_2_/air injection, and light spectrum. For ease of use, the entire system is divided into smaller modules: temperature, gas injection, light system, and sampler modules. Agitation within each vessel is achieved by a combination of magnetic stirring and gas column flow, ensuring homogeneity of the culture. A disadvantage of monoculture microalgae in PBRs is that dissolved oxygen accumulation can reach saturation, which is detrimental to microalgae growth [46]. However, the high oxygen consumption by bacteria in co-cultured algal–bacterial systems has been shown to reduce these negative effects by reducing the amount of dissolved oxygen available [47]. When municipal wastewater was treated in an agitated algal–bacterial PBR, it was estimated that 40–53% and 17–20% of the NH_3_ removal was due to bacterial assimilation and nitrification, respectively [48].

In biofilm reactors, microalgae are immobilised on a surface that acts as a support, resulting in the formation of a continuous layer. This approach offers advantages such as increased cell concentration per volume of medium, simplified harvesting, and reduced or minimal cell presence in the effluent [49]. Algal–bacterial biofilms can be categorised as either stationary or mobile based on the movement of the supporting materials. One of the main advantages of microalgal biofilm reactors over other techniques is that they simplify the extraction and dewatering of algal cells from biofilms. This is due to the ease with which the attached cells can be separated from the surrounding growth medium, eliminating the need for costly separation methods such as filtration, centrifugation, flocculation, or settling/floating of the biomass. In these systems, the biomass must be collected by scraping it from the support medium [50]. The basic principles governing the colonisation of surfaces by motile, photosynthetic microorganisms remain largely unexplored. Regulation of the adhesion properties of *Chlamydomonas* could significantly enhance the efficiency of biofilm reactors by controlling surface colonisation and biofilm formation. Research has shown that surface adhesion of *C. reinhardtii* is flagella-mediated and largely substrate-independent, allowing it to adhere to any type of surface [51]. However, it has been shown that *C. reinhardtii* biofilm adhesion is controlled by the type of light, being activated by blue light and deactivated by red light [52]. The phosphate-hyperaccumulating strain *Chlamydomonas pulvinata* TCF-48 g was tested using the biofilm system to recover phosphate from municipal wastewater and demonstrated a high phosphorus removal rate of 70% [53]. Using *Chlamydomonas* sp. JSC4, this technique has been successfully optimised for the removal of phosphorus, nitrogen, and copper from pig effluent [54].

In biofilm reactors, the medium flows through the bioreactor while the biomass remains attached to a stationary support medium; therefore, the residence time of the algae and bacteria is much longer. This allows algal–bacterial biofilm reactors to operate at higher organic loading rates and shorter hydraulic retention times than suspended growth systems, as slow-growing communities are retained in the reactor. Numerous experiments, both small and large, have been carried out using algae–bacteria biofilm reactors for wastewater treatment [55,56]. Algal–bacteria biofilm reactors can be flushed with CO_2_ to increase biomass productivity or be integrated with other treatment processes to increase the efficiency of wastewater treatment. A biomass productivity of 60 g/m^2^/day has been reported in algae–bacteria biofilm reactors using synthetic wastewater and flushed with 0.5% CO_2_ enriched air [57]. The use of an algae–bacteria biofilm reactor in conjunction with additional reactors offers the potential to refine secondary effluent, further reducing total suspended solids concentrations to below 0.5 mg/L [58].

Encapsulation of microalgae involves the entrapment of microalgae within coating materials, resulting in the formation of beads. This process offers several benefits, such as promoting controlled release, protecting the formation of bioactive compounds, increasing bioavailability, and improving solubility [59]. Various materials such as alginate, chitosan, carrageenan, and polyvinyl have been used to immobilise microalgae [60]. In *Chlamydomonas*, alginate is the most widely used because of its low cost, biocompatibility, transparency, and permeability, which facilitate the diffusion of nutrients and light [61]. In addition, the preparation of alginate beads is a quick and straightforward process that can be easily scaled up [62]. Encapsulation of *C. reinhardtii* in alginate has been successfully used to remove various types of contaminants such as nitrogen, phosphorus, cadmium, lead and mercury [63], or even phenol [64]. With *C. reinhardtii* alginate beads, pore size has been shown to be critical for contaminant removal, with the highest removal efficiency achieved with a gel bead pore size of 3.5 mm [65]. Silica hydrogels have been used to encapsulate *C. reinhardtii* cells and offer some advantages over alginate, such as higher transparency and greater stability against ions and microbial attack. Silica hydrogels encapsulating *Chlamydomonas* have shown potential for applications such as hydrogen production [66]. A disadvantage of alginate encapsulation is its high porosity, which can lead to the release of large molecules. However, it has been found in *C. reinhardtii* that the combination of alginate and silica to form hybrid beads can offer improved properties that overcome this limitation [67]. Single-cell encapsulation involves coating individual cells with metal–phenolic networks to create a mechanical barrier. In microalgae, this method was first applied to *C. reinhardtii*, where it was found that this approach helped to delay the proliferation of the coated cells and effectively promoted flocculation [68]. One advantage of encapsulation is that it can be carried out with several organisms at the same time, a process known as co-immobilisation. In the case of algae–bacteria encapsulation, the bacteria can actually be trapped within the porous structure of the encapsulating material. Some studies have provided microscopic evidence of this phenomenon [69]. Co-immobilisation of *C. reinhardtii* with the acetate-producing cyanobacteria *Synechococcus* sp. PCC 7002 increases the biomass content [70]. Co-immobilisation of *Chlamydomonas* and the cyanobacterium *Lyngbya* sp. on silica hydrogel showed a 92.5% removal of Pb^2+^ from wastewater [71]. Studies have also shown that when *Chlamydomonas* is co-immobilised with the nitrogen-fixing bacterium *Azospirillum brasilense*, there is a mutualistic relationship supported by the exchange of tryptophan and IAA, respectively. This relationship enhances microalgal CO_2_ fixation and biomass production [72].

## 3. Wastewater Types, Composition and Treatment Methods

**Figure 1:**
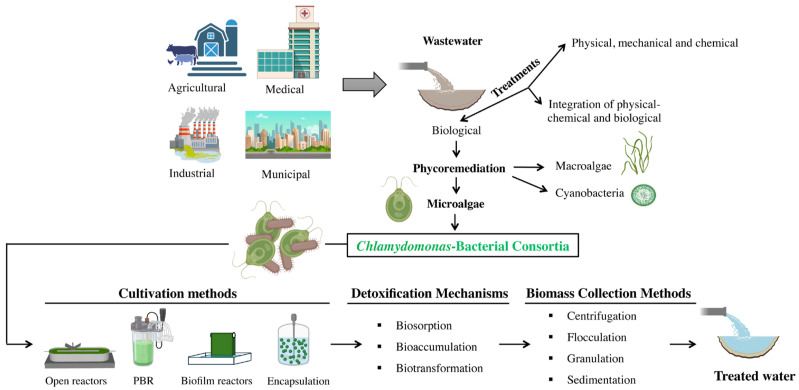
Schematic overview of the wastewater treatment process using *Chlamydomonas*–bacterial consortia.

Water can be polluted by a variety of sources, each with a different intensity. Wastewater can originate from a variety of sources including domestic, commercial, residential, industrial, surface runoff, recreational, institutional, and agricultural ones (Figure 1). The composition of wastewater varies considerably depending on the source and the industrial processes involved and comprises a diverse mixture of organic, inorganic, and synthetic compounds, with carbohydrates, fats, sugars, and amino acids being among the major contaminants [73]. Persistent organic pollutants include chlorinated and aromatic compounds, such as organochlorine pesticides, polycyclic aromatic hydrocarbons, and polychlorinated biphenyls [74]. Inorganic constituents found in wastewater include substances such as sodium, calcium, nitrates, potassium, magnesium, sulphur, bicarbonate, arsenic, heavy metals, chlorides, phosphates, and non-metallic salts [75]. Municipal wastewater contains organic matter, nutrients, pathogens, and chemicals. Agricultural wastewater contains organic matter, pesticides, herbicides, and fertilisers. Dairy effluent typically has a very acidic pH, high concentrations of organic compounds, and high levels of organic and inorganic phosphate and nitrogen, fats, oils, and detergents. Industrial effluent may contain heavy metals, organic chemicals, and oils. Synthetic wastewater is a laboratory-created imitation used for research purposes [76]. Medical wastewater contains antibiotics and antibiotic resistance genes, organic pollutants (e.g., phenol and its derivatives), refractory micropollutants (e.g., triclosan, ibuprofen, diclofenac), and toxic chemicals (e.g., cyanides, chlorinated lignin, dyes) [77]. As a result, each type requires specific treatment due to its unique contaminants.

As anthropogenic activities increase, leading to more complex wastewater compositions, it is essential to develop wastewater treatment methods that are easy to use, effective, and environmentally friendly to reduce water pollution. Traditional wastewater treatment methods include physical, mechanical, chemical, and biological approaches (Figure 1) [78]. Physical methods include sedimentation, screening, and skimming; mechanical methods use filtration techniques; chemical methods include processes such as adsorption, neutralisation, disinfection, precipitation, and ion exchange [79]; and biological methods use microorganisms to break down pollutants [80]. The integration of physico-chemical and biological methods offers a sustainable solution by reducing energy and chemical consumption, cutting costs, and minimising environmental impact [81].

Among the biological methods, phycoremediation, derived from the Greek word for algae ‘phyco’, is an environmentally friendly approach that uses various types of algae, such as microalgae, macroalgae, or cyanobacteria, to purify wastewater by removing pollutants or extracting products from it (Figure 1). Notable applications of phycoremediation include the removal of nutrients and xenobiotic compounds, the reduction of excess nutrients in organic-rich wastewater, the reduction of CO_2_, the treatment of wastewater with heavy metal ions, and the use of algae as biosensors to monitor potentially harmful substances [82]. Phycoremediation not only helps to remove pollutants but also results in the production of algal biomass that can be used to produce various valuable products such as food, fertilisers, pharmaceuticals, and biofuels [83]. *Chlamydomonas* and other algae have been extensively used in wastewater treatment [84,85].

## 4. Main Mechanisms and Molecules Detoxified by *Chlamydomonas*

*Chlamydomonas* is capable of detoxifying several molecules [86]. We will now outline the different mechanisms and types of molecules that can be bioremediated by *Chlamydomonas*. Microalgae have the ability to absorb and degrade pollutants such as heavy metals, hydrocarbons, and pesticides using mechanisms such as biosorption, bioaccumulation and biotransformation [87] (Figure 1). In this study, Bhatia and colleagues explore innovative approaches to wastewater treatment, with a focus on microalgae-based technologies for resource recovery and bioenergy production. The diverse composition of wastewater types is addressed, and advances in cultivation and biomass-harvesting methods are discussed. Biosorption is a passive process in which microalgae act as a biological sorbent to bind and concentrate contaminants. Microalgae use their cell wall and various chemical groups to attract and retain metallic and organic contaminants. The cell wall of *Chlamydomonas* is not made up of cellulose as in plants but of five dense glycoprotein-rich layers [88]. The cell surface of *C. reinhardtii* has a net negative charge, with a zeta potential typically between −10 and −30 mV. This negative surface charge is thought to result from the presence of carboxyl and phosphate groups on the glycoproteins and polysaccharides in the cell wall [89]. The pollutants adhere to the algal membrane and are separated by the presence of receptors that can bind and attract them [90]. The authors explore the use of microalgae for phycoremediation of carcinogenic heavy metals such as arsenic, cadmium, chromium, lead, and mercury, highlighting their efficacy, environmental friendliness, and potential to produce valuable co-products. *Chlamydomonas* has developed defence mechanisms against Hg toxicity, including binding Hg to intracellular ligands such as phytochelatins and sulfhydryl groups to form cumulative metal complexes [91]. Biosorption is indeed a well-studied mechanism for the removal of heavy metals from wastewater. *C. reinhardtii* has demonstrated a high capacity to remove arsenic [92], copper, boron, and manganese [93] by biosorption. In this study, Saavedra and co-workers provide a comparative analysis of four green microalgal species in their uptake of arsenic, boron, copper, manganese, and zinc from monometallic and multimetallic solutions, highlighting their different efficiencies influenced by pH and contact time. It highlights the potential of microalgal biomass as effective biosorbents for these toxic elements, with insights into their interactions as revealed by Fourier Transform Infrared (FTIR) spectra, particularly notable for arsenic and copper. Nickel [94] and uranium [95] have also been detoxified by *Chlamydomonas.* A plant cadmium (Cd) and zinc (Zn) transporter (AtHMA4) was used as a transgene to enhance the ability of *Chlamydomonas reinhardtii* to tolerate exposure to 0.2 mM Cd and 0.3 mM Zn [96]. Microalgae can remove pollutants through a process known as bioaccumulation. The difference between biosorption and bioaccumulation processes lies in the fact that biosorption is a passive process in which microorganisms use their cellular structure to trap pollutants, whereas bioaccumulation is an active process characterised by the accumulation of pollutants in the biomass of microalgae, achieved either by accumulation or intracellular uptake [97]. *C. reinhardtii* has been shown to bioaccumulate several compounds, including o-nitrophenol [98], the herbicide Prometryne [99], and the anti-epileptic drug carbamazepine in *Chlamydomonas mexicana* [100]. *C. mexicana* was found to be more tolerant to carbamazepine than S. obliquus, achieving up to 35% biodegradation and identifying two metabolites by HPLC-MS analysis. This suggests the potential of *C. mexicana* for effective treatment of carbamazepine-contaminated wastewater. Biotransformation involves the degradation of pollutants, either inside or outside cells, with the aid of enzymes [101]. While biosorption and bioaccumulation are not of significant concern, biotransformation poses a greater challenge as the by-products may be more toxic than the original compounds. Some of the pollutants removed by biotransformation by *C. reinhardtii* include polystyrene [102], polycyclic aromatic hydrocarbons such as organophosphorus, and pesticides such as trichlorfon [103]. Selenite uptake by *C. reinhardtii* was investigated. The results indicated that the adsorbed fraction was negligible compared to the absorbed fraction [104]. *C. reinhardtii* was exposed to selenite, selenate, or selenomethionine at different concentrations of H^+^ ions and sulphate. At low sulphate concentrations, selenite was more readily accumulated than selenate and selenomethionine. However, at higher sulphate concentrations, the uptake of selenite was higher than that of selenate, whereas the uptake of selenomethionine remained unchanged. Selenium concentrations measured in *C. reinhardtii* exposed to selenomethionine were 30 times lower than those found in field-collected microplankton under the same laboratory conditions [105]. The efficient biodegradation of the carcinogenic benz(a)anthracene BaA by *C. reinhardtii* CC-503 was investigated. The algae effectively degraded BaA at concentrations up to 10 mg/L within 11 days, producing intermediate metabolites such as isomeric phenanthrene or anthracene, 2,6-diisopropylnaphthalene, and others. Upregulation of genes encodes degradation enzymes such as homogentisate 1,2-dioxygenase [106]. The physiological and proteomic responses of *C. reinhardtii* to long-term exposure to microplastic bisphenol A (BPA) highlight its effects on iron and redox homeostasis and the induction of ferroptosis. The research reveals molecular mechanisms of BPA toxicity and how the algae recover through ROS detoxification and proteomic adjustments and sheds light on potential target genes for the development of efficient strains for microplastic bioremediation [107]. Anti-inflammatory drugs, such as ibuprofen [108], and antibiotics, such as sulfadiazine [109], have also been detoxified by *Chlamydomonas*. Ibuprofen caused severe damage to *Chlamydomonas*, including decreased vitality and metabolic activity, increased ROS levels, disrupted membrane potentials, and induced programmed cell death accompanied by DNA fragmentation. Genetically modified *C. reinhardtii* expressing the cyanase gene from *Synechococcus elongatus* showed the ability to remediate high levels of potassium cyanide, up to 150 mg/L [110]. These findings underline the ability of *Chlamydomonas* to eliminate various substances, highlighting its role in environmental processes and potential applications in detoxification.

## 5. *Chlamydomonas*–Bacterial Consortia for Bioremediation

The use of microalgae–bacteria consortia in detoxification is known to be a promising approach for wastewater treatment [111]. These consortia exploit the synergistic relationship between microalgae and bacteria to efficiently degrade organic matter, remove inorganic compounds, increase biomass production, or improve influent quality, among other benefits [112]. In fact, the use of microalgae–bacteria consortia for bioremediation treatment can offer several advantages over the use of microalgae monocultures alone, such as improved nutrient or antibiotic removal and reduced contamination risk. The combined metabolic activities of microalgae and bacteria reduce the risk of contamination compared to microalgae monocultures, as the diverse microbial community is more resilient to environmental changes and potential invaders [113]. A key interaction between microalgae and bacteria is the exchange of CO_2_ and O_2_. Aerobic bacteria consume the oxygen produced by algal photosynthesis and in turn produce CO_2_, which supports algal growth. Bacteria can play a major role in breaking down complex organic matter, making it more readily available for uptake by microalgae [114]. Microalgae–bacteria consortia show superior performance in removing veterinary antibiotics from synthetic wastewater and pig wastewater in pilot-scale photobioreactors [115].

While there are numerous studies supporting the use of microalgae–bacteria consortia in various wastewater detoxification applications [116], the use of *Chlamydomonas*-based consortia is a relatively unexplored topic with promising results. Different types of bacteria have shown the ability to enhance the detoxification potential of *Chlamydomonas*. The potential of *C. reinhardtii* in consortia with three different bacterial strains (*Stenotrophomonas maltophilia*, *Microbacterium paraoxydans*, and *Paenibacillus lactis*) for the detoxification of phenol-contaminated wastewater was investigated. The results show that the consortium of *M. paraoxydans* and *C. reinhardtii* was very effective in the removal of phenol due to the synergistic interactions between the microalgae and bacteria, which enhance algal growth [117]. A cooperative consortium between *C. reinhardtii* and the bacterium *Methylobacterium oryzae* was also found to result in increased biomass production and inorganic nitrogen removal when grown in urban wastewater [118]. Bacteria play a crucial role in the nitrification and denitrification processes that are essential for the complete removal of nitrogen as gas, and both algae and bacteria also contribute to nitrogen removal through assimilation [119]. As mentioned above, microalgae produce oxygen during photosynthesis, which supports bacterial nitrification while reducing the energy required for culture aeration. *C. reinhardtii* in consortia with bacteria has been shown to remove nitrogen species from municipal wastewater, achieving up to 80% removal of dissolved organic nitrogen (DON) [120]. In a study using animal effluent collected from two different sources, a feedlot effluent storage tank and a sheep effluent storage lagoon, the results showed that between 36 and 79% of the initial DON was removed by the *Chlamydomonas*–bacteria consortia [121]. *Acidithiobacillus ferrooxidans* and *Acidithiobacillus thiooxidans* in consortia with *Chlamydomonas* have been shown to reduce the contamination of sediments with heavy metals (Cu, Pb, Zn, Mn, Cd, As) [122]. The co-culture of *C. reinhardtii* with different bacterial strains has allowed the identification of bacterial species such as *Sphingobium yanoikuyae*, capable of degrading hydrocarbons and aromatic compounds [123], *Acidovorax* sp. *A16OP12*, which can use common environmental pollutants as a carbon source [124], and *Microbacterium* sp. are capable of removing heavy metals and antibiotics [125].

## 6. *Chlamydomonas*–Bacterial Consortia for Biomass and Bio-Product Generation

### 6.1. Biomass

Microalgae biomass is highly valued in a bio-based economy, being used for the sustainable production of various products such as fuel, food, energy, pharmaceuticals, and others [126]. The use of microalgal biomass grown in urban wastewater can be limited by potential contamination, variability in composition, scalability challenges, regulatory hurdles, and economic feasibility. Microalgae can contain contaminants and their composition can vary, making consistent quality difficult. Scaling up production and meeting regulatory standards are challenges, while economic viability may be uncertain compared to alternative biomass sources or waste treatment methods. Overcoming these limitations requires further research to fully realise the potential of microalgal biomass production in urban wastewater systems. Microalgae have several advantages in biomass production: they are more productive per unit area than any other plant system, do not compete for arable land, and can be grown throughout the year [127]. Microalgal biomass is a versatile resource that offers a wide range of applications through the extraction of specific compounds for various purposes. The composition of the biomass, including proteins, lipids, and carbohydrates, as well as active compounds, is significantly influenced by the strains of microalgae and the cultivation conditions to which they are exposed [128]. Various approaches have been explored to obtain microalgal biomass enriched in specific biomolecules. Studies on increasing biomass production by co-cultivation of *Chlamydomonas* and bacteria have shown promising results. Next, we will present studies in which an increase in biomass production is achieved through the interaction of bacteria and *Chlamydomonas*. The vitamin B12-auxotrophic *Chlamydomonas* strain was able to increase its biomass when co-cultured with B12-producing bacteria, including the rhizobium *Mesorhizobium loti* or even an *E. coli* strain engineered to produce and release B12 [129]. *Methylobacterium* spp. have excellent biotechnological potential in agriculture because they produce phytohormones, promote plant growth through N_2_ fixation, and provide protection against pathogens and pollutants [130]. The co-culture of *Mesorhizobium oryzae* and *C. reinhardtii* in ethanol-containing media significantly increased biomass production, with a potential increase of up to 700%. The crucial metabolic aspect of this association depended on the bacterial conversion of ethanol to acetate, which supported the heterotrophic growth of *C. reinhardtii* [118]. *Methylobacterium aquaticum* has been shown to enhance the biomass production of *C. reinhardtii* in a proline-rich medium by transferring part of the NH_4_^+^ obtained through its metabolism to the alga. In turn, *C. reinhardtii* donates part of its fixed carbon in the form of glycerol to *M. aquaticum*, which uses it as a carbon source [21].

Diazotrophs are microorganisms that have the ability to convert atmospheric nitrogen into bioavailable forms such as ammonia. The microalgae, including *Chlamydomonas*, have shown remarkable interactions with various types of nitrogen-fixing organisms [131]. *Azotobacter* spp., which are free-living diazotrophs, have been widely used as biofertilizers, effectively increasing the yield of various crops [132]. Several *Azotobacter* species, including *A. chroococcum, A. beljerinckii, A. agilis*, and *A. vinelandii*, have been shown to enhance *C. reinhardtii* biomass production by transferring some of the fixed nitrogen to *Chlamydomonas* [133]. On the other hand, researchers have also observed that *C. reinhardtii* can support the growth of *Azotobacter* by transferring some of the carbon fixed by photosynthesis, although the specific compounds involved in this process have not yet been identified [134]. This *Chlamydomonas–Azotobacter* mutualism could be of great biotechnological importance, as it would enable the production of large amounts of biomass using only N_2_ and CO_2_ from the air, without the need to add organic nitrogen and carbon sources, which would reduce the economic viability of the process.

### 6.2. Biofuels

Biofuels are derived from renewable biological sources such as plants or plant-derived materials and include biodiesel, bioethanol, biogas, and biohydrogen. First-generation biofuels are produced from food crops, second-generation biofuels are produced from non-food sources such as waste, and third-generation biofuels are produced from sources that do not compete with arable land. Biofuels produced from algae are third-generation biofuels [135]. These biofuels can be produced through transesterification, photosynthesis-mediated microbial fuel production, and other thermochemical and biochemical conversions [136]. Several approaches have been taken to increase biofuel production through the co-cultivation of microalgae and bacteria [137], with studies focusing mainly on biohydrogen production in *Chlamydomonas*.

#### 6.2.1. Biohydrogen

Biohydrogen refers to hydrogen produced by living organisms such as bacteria, cyanobacteria, and algae [138]. The first evidence that *Chlamydomonas* had the ability to produce H_2_ came from experiments with *C. moewusii* under anaerobic conditions [139]. In biological processes, hydrogenases are the enzymes responsible for hydrogen production [140]. *Chlamydomonas* has two different hydrogenases, which have been the subject of extensive research with the primary aim of finding ways to increase the efficiency of hydrogen production [141]. Hydrogenases can use two processes to obtain the energy for the reduction of H^+^ to H_2_: they can either use energy from light, a process known as biophotolysis, or oxidise organic compounds such as starch, a process known as dark fermentation. The main challenge in using *C. reinhardtii* for hydrogen production is the rapid inactivation of both hydrogenases by oxygen, especially since oxygen is produced during photosynthesis. Therefore, the first evidence of *C. reinhardtii*’s ability to produce H_2_ was obtained under anaerobic conditions [142]. The use of sulphur-deprived *C. reinhardtii* was the first successful strategy to demonstrate significant and consistent hydrogen production under aerobic conditions [143]. This happens because the lack of sulphur inhibits protein synthesis, which in turn disrupts photosynthesis and the production of oxygen.

**Figure 2:**
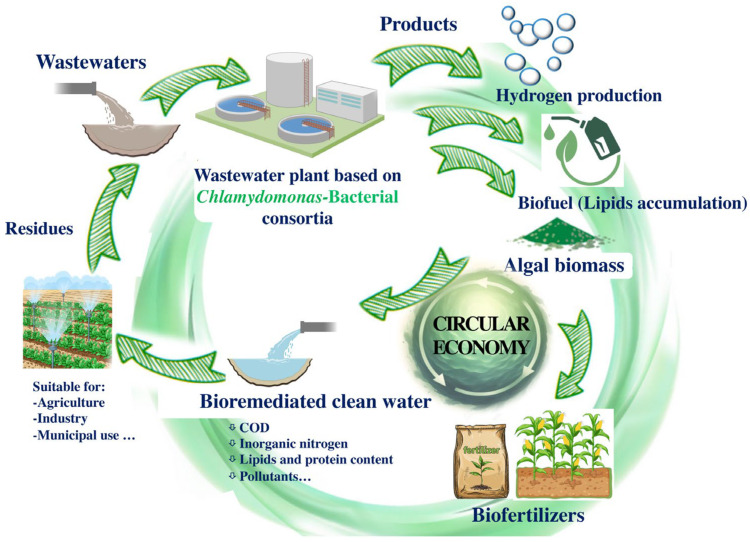
Schematic overview of a hypothetical model based on the concept of a circular economy using *Chlamydomonas*–bacteria consortia in wastewater treatment plants. This treatment results in algal biomass that can be converted into bio-hydrogen biofuel through lipid accumulation and biofertilizers.

*Chlamydomonas*–bacteria consortia have opened a new window to improve hydrogen production (Figure 2). Several studies have successfully used different consortia between *C. reinhardtii* and different bacterial partners and analysed the factors behind the improved hydrogen production [144]. One of the main reasons why the co-cultivation of *Chlamydomonas* and bacteria enhances hydrogen production is that the bacteria consume oxygen, preventing the inhibition of hydrogenase, as observed when *Chlamydomonas* was co-cultured with *A. chroococcum* [145] or with *Bradyrhizobium japonicum* [146]. In the co-culture of *Chlamydomonas* with *P. putida, P. stutzeri, Rhizobium etli,* and *E. coli*, an increase in *Chlamydomonas* hydrogen production was also observed. This increase was attributed not only to the bacterial consumption of oxygen but also to the bacterial production of acetic acid from sugars, which *Chlamydomonas* uses as a carbon source [147,148]. In the co-culture of *C. reinhardtii* and *Methylobacterium oryzae*, the improved hydrogen capacity of *Chlamydomonas* was due to the ability of *M. oryzae* to oxidise ethanol to acetate, which supports the heterotrophic growth of *Chlamydomonas* [118]. In the co-culture of *C. reinhardtii* and *Mesorhizobium sangaii*, the addition of different nitrogen compounds, such as NaNO_2_, NaNO_3_, and NH_4_Cl, resulted in enhanced photobiological hydrogen production compared to monocultures. Specifically, the addition of 3 g/L NaNO_2_ resulted in a maximum H_2_ production of 226.98 μmol/mg chlorophyll, which was 5.2 times higher than that of the pure algal culture [149].

A very promising approach by *Chlamydomonas*–bacterial consortia is the production of hydrogen from wastewater (Figure 2). Although the production of hydrogen from wastewater is very challenging, research into hydrogen production from waste decomposition is an interesting avenue with significant scale-up potential. Considering that *Chlamydomonas* serves as a model microalga for biohydrogen production and that bacteria contribute to the enhancement of hydrogen production, it is logical to explore the capacity of *Chlamydomonas*–bacteria consortia for biohydrogen production alongside wastewater detoxification [150]. In fact, biohydrogen production by three microalgae, including *C. reinhardtii*, has led to a novel approach to autotrophic denitrification using hydrogen-consuming denitrifiers [151]. Nitrate contamination of drinking water is a major environmental problem. Autotrophic denitrification, which uses microorganisms to convert nitrate into harmless gases, is a promising alternative, but an electron donor is needed to sustain the process. In this sense, the hydrogen produced by *Chlamydomonas* in co-culture with water-consuming denitrifiers acts as an electron donor, providing a sustainable, innovative, and environmentally friendly solution to nitrate pollution [151]. A mixed culture of *Chlamydomonas–Rhizobium* consortia has also been demonstrated for coupled hydrogen and biogas production [152]. After the hydrogen production stage, the *Chlamydomonas-Rhizobium* biomass was used for biogas production in the second stage of the process. This study demonstrates that the *Chlamydomonas–Rhizobium* consortium can produce hydrogen without the need for sulphur removal, which is typically required for biohydrogen production by *Chlamydomonas* monoculture.

#### 6.2.2. Lipids

Triacylglycerols (TAGs) are key lipids in microalgae for biofuel production. Oleaginous microalgae, which are rich in TAGs, can be converted into biodiesel through transesterification, a process that converts TAGs into fatty acid methyl esters (FAMEs), the key components of biodiesel [127]. Using *Chlamydomonas* sp. JSC4, a direct transesterification process was employed, resulting in almost 100% biodiesel production in a single step [153]. Since biodiesel production is closely linked to the amount of lipids and TAGs, various strategies have been explored to increase their production in *Chlamydomonas* and its consortia. Some studies have focused on elucidating the functions of key genes involved in lipid and TAG production. A mutant strain of *C. reinhardtii* lacking phospholipase showed an increase in TAG content of up to 190% [154]. The target of rapamycin (TOR) is essential for the regulation of cell growth. Studies have shown that *C. reinhardtii* mutants deficient in TOR show an increase in TAG production [155]. In *C. reinhardtii*, mutation of ACX2, which encodes a member of the acyl-CoA oxidase responsible for the first step of peroxisomal fatty acid beta-oxidation, resulted in a 20% increase in lipid accumulation [156]. Down-regulation of the phosphoenolpyruvate carboxylase gene in *C. reinhardtii* resulted in a 74.4% increase in lipid content [157]. The strategy of heterologous gene overexpression in *Chlamydomonas* has been successful in increasing TAG content. Specifically, heterologous expression of the *Dunaliella tertiolecta* fatty acyl-ACP thioesterase in *C. reinhardtii* results in increased lipid production [158]. Heterologous expression of the glycerol-3-phosphate acyltransferase from *Lobosphaera incisa* in *C. reinhardtii* increases TAG production [159]. Introducing the diacylglycerol acyltransferase from *Saccharomyces cerevisiae* into *C. reinhardtii* increased fatty acid content by 22% and TAG content by 32% [160]. Overexpression of the ferredoxin gene *PETF* in *C. reinhardtii* resulted in increased lipid content [161]. Overexpression acetyl-CoA synthetase led to a 2.4-fold increase in TAG accumulation [162]. Genetic modification of *Chlamydomonas* sp. JSC4 in the gene encoding the starch-debranching enzyme promotes carbohydrate degradation and redirects carbon resources into lipids, resulting in a 1.46-fold increase in lipid production [163]. The synthesis of starch and lipids competes for carbon skeletons; therefore, inhibiting starch synthesis is another strategy used to increase TAG production. In this context, knocking out ADP-glucose pyrophosphorylase in *C. reinhardtii* resulted in a tenfold increase in TAG content [164].

In studies of lipid production in *Chlamydomonas* co-cultures, a high lipid content was observed when a consortium of *Chlamydomonas* and cyanobacteria was cultured in wastewater from a dairy farm. The consortium was found to be able to remove more than 98% of the nutrients from the wastewater, achieving high biomass production and algal lipid content that could be converted into biodiesel [165]. It has been observed that immobilising a co-culture of *C. reinhardtii* with the acetate-producing cyanobacteria *Synechococcus* sp. PCC 7002 increases the microalgal lipid yield [70]. The addition of *A. chroococcum* to the *C. reinhardtii* culture significantly increased lipid accumulation and productivity compared to the traditional nitrogen deprivation condition to increase lipid accumulation, making it an efficient strategy to increase lipid production [166]. Co-culturing *Chlamydomonas* sp. in the presence of the floc-forming bacterium *Bacillus infantis* showed that the co-cultures had higher microalgal biomass and lipid content compared to the axenic culture [167]. The co-culture of *C. reinhardtii* in municipal and swine wastewater effluents exhibited enhanced algal growth, biomass production, and lipid accumulation in the presence of indigenous bacteria [168].

### 6.3. Biofertilizers

Microalgae act as biofertilizers and biostimulants, enhancing plant growth and enriching soil nutrients, reducing the need for chemical fertilisers and supporting sustainable agriculture [169]. Despite their abundance in natural soil ecosystems, *Chlamydomonas* species have been largely overlooked and underutilised in agricultural practises. In this context, lyophilised powders of *C. reinhardtii* have been found to have a beneficial effect on maize plant growth, producing bioactive compounds that act as biostimulants, improving plant growth, yield, quality, and crop performance [170]. *Chlamydomonas sajao* has been shown to improve soil physical properties such as aggregation and stability, thereby improving soil structure and nutrient retention [171]. Research into the effect *of Chlamydomonas applanata* M9V as a biofertilizer on wheat growth showed that both live and dead forms of the microalgae outperformed a given amount of chemical fertiliser in terms of fresh weight, plant height, carotenoids, and chlorophyll content [172]. Application of live *Chlamydomonas* cells by the soil drench method significantly increased leaf size, fresh weight, shoot length, pigment content, and number of flowers in *Medicago truncatula* [173]. Acid-hydrolysed dry biomass of *C. reinhardtii* enhanced the nitrogen, phosphorus, and carotenoid levels in *Solanum lycopersicum* [174]. Extracts from the biomass of *Chlamydomonas* sp. demonstrated auxin-like activity, leading to an increased number of roots in cucumber plants [175].

The importance of microalgae in the plant microbiome has only recently been recognised, and their use to improve soil fertility, conserve water, and promote plant growth is now emerging as a promising strategy for sustainable agriculture [176]. The plant hormone IAA, known as auxin, is a key signalling molecule involved in the regulation of plant growth and physiology [177]. Microalgae can release L-tryptophan, which can be converted by bacteria into IAA to stimulate algal growth [178]. In this regard, *Chlamydomonas* has the ability to release L-tryptophan, which serves as a suitable substrate for symbiotic microbes to produce IAA [179]. It has also been shown that *Chlamydomonas* can also synthesise IAA from L-tryptophan, an activity mediated by the enzyme L-amino acid oxidase [22]. Interestingly, elevated IAA levels inhibit *Chlamydomonas* cell proliferation and chlorophyll degradation. However, in a consortium of *C. reinhardtii* with the plant growth-promoting bacterium *Methylobacterium aquaticum*, these inhibitory effects were alleviated. These results may have significant agricultural implications, as *Chlamydomonas* and *Methylobacterium* spp. coexist in the plant rhizosphere [180]. Their ability to regulate IAA levels could influence plant health and could be used to improve crops in sustainable agriculture.

## 7. Conclusions and Future Perspectives

This review aims to highlight the considerable biotechnological potential of the microalga *Chlamydomonas* and its bacterial consortia, particularly in the fields of bioremediation, biofertilization, and the production of high-value products such as biofuels and hydrogen. Various *Chlamydomonas*–bacterial consortia have been shown to be effective in treating various types of wastewater, including municipal, agricultural, and industrial, by significantly reducing contaminants and facilitating resource recovery. Importantly, the biomass derived from the cultivation of microalgal–bacteria consortia serves as a valuable feedstock for the production of other bioproducts (Figure 2). Economically, it would be highly advantageous to use this biomass from the bioremediation processes for bioremediation. The use of *Chlamydomonas* and its consortia in agriculture, particularly as biofertilizers and biostimulants, has significant potential to improve soil structure, enhance nutrient retention, and promote plant growth, thereby supporting sustainable agricultural practises and reducing dependence on chemical fertilisers. In addition, various cultivation methods such as open reactors, closed photobioreactors, and biofilm reactors have been optimised to increase the efficiency of *Chlamydomonas*–bacteria co-cultivation.

However, the use of biomass and water from wastewater treatment faces several inherent challenges. These include the presence of xenobiotic residues and heavy metals in the biomass, the scalability of biomass production, and contamination with bacteria, fungi, and viruses, all of which limit their widespread application. Future improvements and optimisations of these processes are crucial to address these drawbacks, with the ultimate goal of integrating them into a circular economy model that reduces waste, reuses materials, and minimises environmental and economic impacts. Ongoing research is expected to improve the efficiency of wastewater bioremediation by focusing on optimising consortium composition and cultivation methods to maximise contaminant removal and biomass production. Advances in genetic engineering and synthetic biology could further enhance the metabolic capabilities of *Chlamydomonas*, enabling the production of high-value compounds and biofuels. In addition, studying the symbiotic relationships between *Chlamydomonas* and different bacterial species may reveal new mechanisms for nutrient cycling and environmental sustainability. Integrating these consortia into circular economy models holds promise for sustainable agricultural practises, bioenergy production, and environmental remediation, contributing to a greener and more sustainable future (Figure 2). In conclusion, advancing the cultivation and application of *Chlamydomonas*–bacteria consortia in various biotechnological fields offers significant economic and environmental benefits. Future research should focus on overcoming current limitations and exploring new avenues for sustainable use.

## Data Availability

All data required to evaluate the conclusions of this paper are included in the main text.
